# MMP9 regulates osteogenesis and MMP2 expression through the TGF-β1/SMAD2/3 signaling pathway in lipopolysaccharide-induced osteoblasts

**DOI:** 10.1590/1414-431X2026e15172

**Published:** 2026-03-30

**Authors:** Lei Li, Yang Yang, Ximei Lu, Hui Li

**Affiliations:** 1 Department of Ultrasound, Qingdao 8th People's Hospital, Qingdao, China; 2 Department of Emergency Medicine, Qingdao 8th People's Hospital, Qingdao, China

**Keywords:** MMPs, Inflammation, Osteogenesis, Gene regulation

## Abstract

Matrix metalloproteinases (MMP) act as effectors and regulators in normal growth and development as well as in pathological processes. The gelatinases MMP2 and MMP9 exhibit similar structures and biological reactions. The purpose of this study was to clarify the relationship between MMP9 and MMP2 in lipopolysaccharide (LPS)-induced osteoblasts. MC3T3-E1 cells were pretreated with or without TGF-β1 inhibitor (20 nM) and SMAD2/3 inhibitor (20 nM) for 1 h, and then with pcDNA3.1-mMMP9 (0.8 μg/mL) for 48 h. Quantitative real-time PCR, western blot, and immunocytochemistry were performed to detect MMP2, TGF-β1, and/or SMAD2/3 expression. Luciferase reporter assay and electrophoretic mobility shift assay (EMSA) were performed to examine the regulatory effect of SMAD2/3 on *MMP2* gene transcription. RUNX2, OSX, ALP, type I collagen, and OCN expression was detected in LPS (20 μg/mL)-stimulated MC3T3-E1 cells after MMP9 treatment. MMP9 activated the expression of TGF-β1 and phosphorylation of SMAD2/3. Phosphorylated SMAD2/3 translocalized into nuclei to bind to SMAD-binding elements in the promoter of the *MMP2* gene, inhibiting *MMP2* gene transcription. Additionally, MMP9 increased RUNX2, OSX, ALP, COL I, and OCN expression in LPS-induced MC3T3 cells. MMP9 may regulate osteogenesis through TGF-β1-SMAD2/3/-MMP2 signaling during the inflammation process.

## Introduction

Matrix metalloproteinases (MMPs) are described as calcium-dependent zinc endopeptidases. It has been demonstrated that they participate in the degradation and remodeling of the extracellular matrix (ECM) in normal growth and development as well as in pathological processes (1).

In addition to the role of degrading the ECM, MMPs also recruit osteoclasts to the region where bone resorption occurs and determine the time and location of bone resorption (2,3). Additionally, it has been reported that MMPs are important for osteogenesis. Vu et al. (4) performed MMP9 knockout and subsequently found that MMPs affected bone formation when they observed an abnormal pattern of skeletal growth plate ossification. Mosig et al. (5) found that the loss of MMP2 resulted in decreased bone mineralization, and they demonstrated that MMP2 played a direct role in early skeletal development and bone cell proliferation. MMPs also function as effectors and regulators in the inflammatory response. McQuibban et al. (6) found that, when MMP2 cleaved MCP-3, there was decreased inflammation due to the general chemokine antagonist that resulted. Zhang et al. (7) found that MMP9 inhibited pro-inflammatory cytokine expression and increased osteopontin (OPG) and osteocalcin (OCN) expression in lipopolysaccharide (LPS)-stimulated cells.

MMPs are stringently regulated at multiple levels. Most MMPs are synthesized and secreted from cells as proenzymes, and they are activated by proteinases or *in vitro* by chemical agents (8). MMP zymogens are activated and regulated by MMPs themselves. Sato et al. (9) demonstrated that MMP2 was activated by MMP14, which is a membrane-type (MT)-MMP. Knäuper et al. (10) found that MMP14 activated MMP13 at the cell surface. Dreier et al. (11) found that pro-MMP9 was activated by MMP3 or a MMP14/MMP13 cascade in osteoarthritic chondrocytes. Steenport et al. (12) reported that MMP1 and MMP3 regulated the expression of MMP9 in mouse macrophages. The structures and biological reactions of MMP2 and MMP9 are similar to those of collagenase (13). Wan et al. (14) found that MMP2 expression increased after MMP9 abrogation in mouse apical periodontitis, suggesting that the expression of MMP2 was possibly regulated by MMP9.

Based on the evidence presented, we hypothesized that MMP9 modulates MMP2 expression under inflammatory conditions to promote osteogenic differentiation. Given the well-established role of the TGF-β/SMAD signaling pathway in osteogenesis, which regulates both osteoblast and osteoclast activities and plays a critical role in bone remodeling (15,16), we aimed to investigate whether MMP9 influences MMP2 via the TGF-β/SMAD axis in LPS-stimulated pre-osteoblasts. To test this hypothesis, we conducted a series of experiments, including western blot (WB), fluorescence microscopy, quantitative real-time PCR, electrophoretic mobility shift assay (EMSA), and luciferase reporter assays. Our findings may help elucidate the complex regulatory network of MMPs during inflammation and provide new insights into the molecular mechanisms underlying inflammation-associated osteogenesis.

## Material and Methods

### Antibodies and reagents

Anti-phospho-SMAD2 and anti-phospho-SMAD3 antibodies for immunofluorescence were purchased from Abcam (UK). The anti-phospho-SMAD2, anti-phospho-SMAD3, anti-SMAD2, anti-SMAD3, and anti-RUNX2 antibodies for WB were purchased from Cell Signaling Technology (USA). The anti-SMAD2 and anti-SMAD3 antibodies for EMSA were purchased from Cell Signaling Technology. The anti-TGF-β1 antibody was purchased from Proteintech (USA). The anti-MMP2, anti-OCN, anti-COL I, anti-RUNX2, and anti-GAPDH antibodies were purchased from Abcam. The anti-OSX antibody was purchased from Affbiotech (USA). The anti-ALP antibody was purchased from Proteintech Group, Inc. (China). MMP9 inhibitor (MMP-9-IN-1) and transforming growth factor-β1 (TGF-β1) inhibitor (Disitertide TFA) were purchased from MCE (USA). SMAD2/3 inhibitor (TP0427736 HCl) was purchased from Selleck (USA).

### Plasmids

mMMP9, mSMAD2, and mSMAD3 expression plasmids were constructed by encoding full-length mMMP9, mSMAD2, and mSMAD3 into the pcDNA3.1 vector. All constructs were verified by DNA sequencing.

### Cell culture

MC3T3-E1 osteoblastic cells were obtained from HYcell Biotechnology (China) and cultured in α-minimum essential medium (MEM, Hyclone, USA) containing 10% fetal bovine serum (FBS, Hyclone), penicillin (100 U/mL), and streptomycin (100 mg/mL) (Hyclone) at 37°C in a humidified atmosphere with 5% CO_2_ and 5% air. The medium was refreshed every two days.

### Quantitative real-time PCR (qRT-PCR) analysis

TRIpure Total RNA Extraction Reagent (ELK Biotechnology, China) was used to extract total RNA. First-strand cDNA was synthesized using the Reverse Transcription System (ELK Biotechnology) as directed by the manufacturer. *MMP9*, *MMP2*, *SMAD2*, *SMAD3*, *TGF-β1*, and *RUNX2* genes were quantified using the StepOne™ Real-Time PCR System (Life Technologies, USA). Internal normalization control was carried out using glyceraldehyde-3-phosphate dehydrogenase (*GAPDH*). The primers used for qRT-PCR are listed in Table 1. The expression of each gene was determined using the 2-ΔΔCt method (17). The gene expression ratio from three independent experiments is reported as means±SD.

**Table 1:** Oligonucleotide primer sequences used in qRT-PCR.

Gene	Sequence (5′-3′)
*MMP9*	
Forward	AAGGGTACAGCCTGTTCCTGGT
Reverse	CTGGATGCCGTCTATGTCGTCT
*MMP2*	
Forward	GAATGCCATCCCTGATAACCT
Reverse	GCTTCCAAACTTCACGCTCTT
*TGF-β1*	
Forward	AGAGCCCTGGATACCAACTATTG
Reverse	TGCGACCCACGTAGTAGACG
*SMAD2*	
Forward	TGGGGAAGTGTTTGCTGAGTG
Reverse	TGTCTGCCTCCGATATTCTGC
*SMAD3*	
Forward	TCCCCAGCACACAATAACTTG
Reverse	CCGATGTAGTAGAGCCGCAC
*RUNX2*	
Forward	CGCCACCACTCACTACCACAC
Reverse	TGGATTTAATAGCGTGCTGCC
*OSX*	
Forward	GACTACCCACCCTTCCCTCAC
Reverse	CCCACCAAGGAGTAGGTGTGT
*ALP*	
Forward	TGACTACCACTCGGGTGAACC
Reverse	TGATATGCGATGTCCTTGCAG
*COL I*	
Forward	CTGACTGGAAGAGCGGAGAG
Reverse	CGGCTGAGTAGGGAACACAC
*OCN*	
Forward	GCAGGAGGGCAATAAGGTAGTG
Reverse	CCATAGATGCGTTTGTAGGCG
*GAPDH*	
Forward	TGAAGGGTGGAGCCAAAAG
Reverse	AGTCTTCTGGGTGGCAGTGAT

### Western blot analysis

Cells were lysed with lysis buffer (ASPEN, China) and centrifuged at 16,000 *g* for 10 min at 4°C. The total protein in the supernatant was measured using a BCA Protein Assay Kit (ASPEN). Thirty micrograms protein was resolved by 10% sodium dodecyl sulfate-polyacrylamide gel electrophoresis (SDS-PAGE) gels (ASPEN). Proteins were electrophoresed and transferred to nitrocellulose membranes (Millipore, USA). The membranes were blocked with 5% nonfat milk (ASPEN) for 1 h at room temperature. The samples were probed with anti-GAPDH (1:10000; Abcam), anti-MMP9 (1:500; Abcam), anti-MMP2 (1:1000; Abcam), anti-phospho-SMAD2 (1:500; CST, USA), anti-SMAD2 (1:1000; CST), anti-phospho-SMAD3 (1:500; CST), anti-SMAD3 (1:1000; CST), anti-TGFβ1 (1:1000; Proteintech), anti-OSX (1:1000; Affbiotech, USA), anti-OCN (1:500; Abcam), anti-COL I (1:500; Abcam), anti-ALP (1:3000; Proteintech Group, Inc), and anti-RUNX2 (1:1000; CST) antibodies. A goat anti-rabbit IgG conjugated with horseradish peroxidase (HRP) (1:10000; ASPEN) was used for detection.

### Immunocytochemistry

Cells were cultured on glass coverslips in 6-well plates and fixed in 4% paraformaldehyde for 10 min. Fixed cells were rinsed twice in phosphate-buffered saline (PBS) and permeabilized with 0.1% Triton X-100 for 5 min. Cells were then incubated in 2.5% bovine serum albumin (BSA)/PBS for 1 h, followed by overnight incubation with a primary antibody (phospho-SMAD2, 1:200, Abcam; phospho-SMAD3, 1:75, Abcam) at 4°C. Cells were then washed three times in wash buffer and incubated for 1 h with a goat anti-rabbit Dylight 488-conjugated secondary antibody. The coverslips were inverted and mounted on glass microscope slides using 4',6-diamidino-2-phenylindole (DAPI) mounting medium, and the cells were visualized with a Nikon Eclipse Ti-S inverted fluorescence microscope (Nikon, Japan).

### Electrophoretic mobility shift assay (EMSA)

According to Chen et al. (18), nuclear extracts (5-10 μg) were preincubated for 5 min at room temperature in a total volume of 20 μL of binding buffer (10 mM Tris-HCl, pH 7.5, 50 mM NaCl, 1 mM EDTA, 1 mM dithiothreitol (DTT), 5% glycerol) containing 2 μg of poly(dIdC). Afterward, ^32^P-end-labeled DNA fragments (1 ng) were added, and the solution was incubated for an additional 20 min. In competition binding reactions, unlabeled DNA fragments in 100-fold molar excess of the labeled DNA probes were added to the reaction. The products of the DNA-protein reaction were separated by electrophoresis on a non-denaturing 5% polyacrylamide gel in 1× TBE buffer. DNA-protein complexes as well as the unbound DNA probe were visualized on X-ray film. For gel mobility super-shift analysis, an anti-SMAD3 antibody (CST) was preincubated with nuclear extracts 10 min prior to adding the radiolabeled probe.

### Luciferase reporter assay

In 24-well plates, cells were transfected with pGL3-Basic empty vector or pGL3-Luc-mMMP2-promoter-wt or pGL3-Luc-mMMP2-promoter-muts (mut1, mut2, mut1,2) and the pRL-TK *Renilla* luciferase expression vector as an internal control as well as pcDNA3.1-SMAD2, pcDNA3.1-SMAD3, or pcDNA3.1 using Lipofectamine 2000. After 48 h, cells were collected and lysed in passive lysis buffer. The Dual Luciferase Reporter Assay System (Beyotime, USA) was used to perform the luciferase assay as directed by the manufacturer. Firefly and *Renilla* luciferase activities were determined using a Glomax Luminometer (Promega, USA). Luciferase expression was normalized against *Renilla* luciferase expression to determine the relative luciferase activity.

### Alkaline phosphatase (ALP) staining

MC3T3-E1 cells were treated with pcDNA3.1 (control group) or pcDNA3.1-mMMP9 (MMP9 overexpression group) at a final concentration of 0.8 μg/mL using Lipofectamine 2000 transfection reagent (Thermo, USA). After 48 h, cells were stimulated with or without 20 μg/mL LPS (eBioscience, USA) and cultured in calcifying medium (α-MEM supplemented with 10% FBS and 100 IU/mL penicillin), streptomycin (100 μg/mL), 50 μg/mL ascorbic acid, and 10 mM sodium β-glycerophosphate) at 37°C for 7 days. After washing with PBS, cells were fixed in 4% paraformaldehyde for 20 min and then stained with ALP (Aspen Biological, China) according to the manufacturer's instructions.

### Alizarin red S (ARS) staining

MC3T3-E1 cells were treated with pcDNA3.1 (control group) or pcDNA3.1-mMMP9 (MMP9 overexpression group) at a final concentration of 0.8 μg/mL using Lipofectamine 2000 transfection reagent (Thermo). After 48 h, cells were stimulated with 20 μg/mL LPS (eBioscience) and cultured in calcifying medium at 37°C for 21 days. Cells were fixed in 95% methanol for 45 min at 4°C followed by washing with H_2_O. The cultures were then stained with 0.5% ARS (Sigma, USA) for 30 min at 37°C, washed with H_2_O, and observed under microscope.

### Statistical analysis

Statistical analysis was conducted using SPSS 23.0 statistics software (IBM, USA) with a two-tailed *t*-test or ANOVA based on results from three independent experiments. P<0.05 was considered statistically significant.

## Results

**Figure 1:**
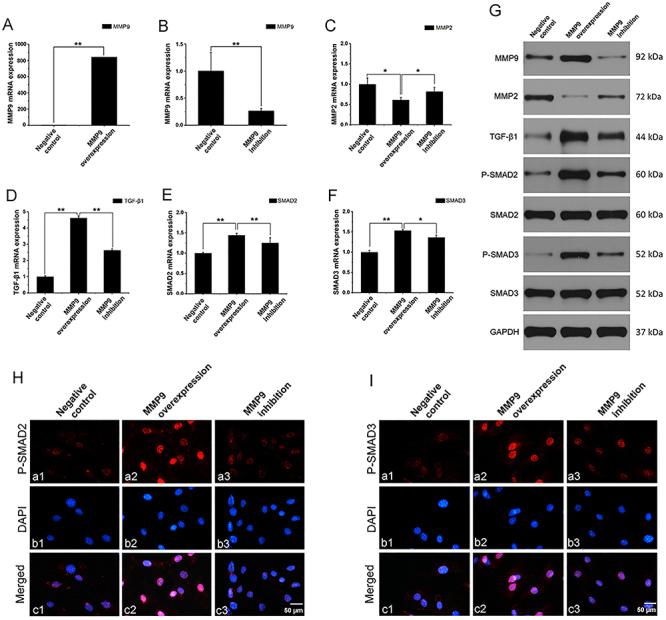
MMP9 regulated MMP2, TGF-β1, SMAD2, and SMAD3 expression in MC3T3-E1 cells. MC3T3-E1 cells were pretreated with or without MMP9 inhibitor for 1 h. After that, cells were transfected with pcDNA3.1 or pcDNA3.1-mMMP9 and cultured for 48 h. mRNA expression of (**A** and **B**) MMP9, (**C**) MMP2, (**D**) TGF-β1, (**E**) SMAD2, and (**F**) SMAD3 was measured by qRT-PCR. (**G**) Protein expression of MMP9, MMP2, TGF-β1, SMAD2, SMAD3, phospho-SMAD2, and phospho-SMAD3 was measured by western blot. Immunofluorescence analysis of (**H**) phospho-SMAD2 and (**I**) phospho-SMAD3 in MC3T3 cells; scale bar 50 μm. Data are representative of three independent experiments and are reported as means±SD. *P<0.05, **P<0.01; *t*-test or ANOVA.

To systematically investigate the role of MMP9 in osteogenic differentiation, we first aimed to establish its fundamental function under normal physiological conditions. Thus, the experiments presented in Figures 1-3 were performed without any inflammatory stimulus. After elucidating this baseline mechanism, we proceeded to examine whether and how this regulatory function is modulated in an inflammatory context, modeled by LPS treatment, as shown in Figure 4.

**Figure 2 f02:**
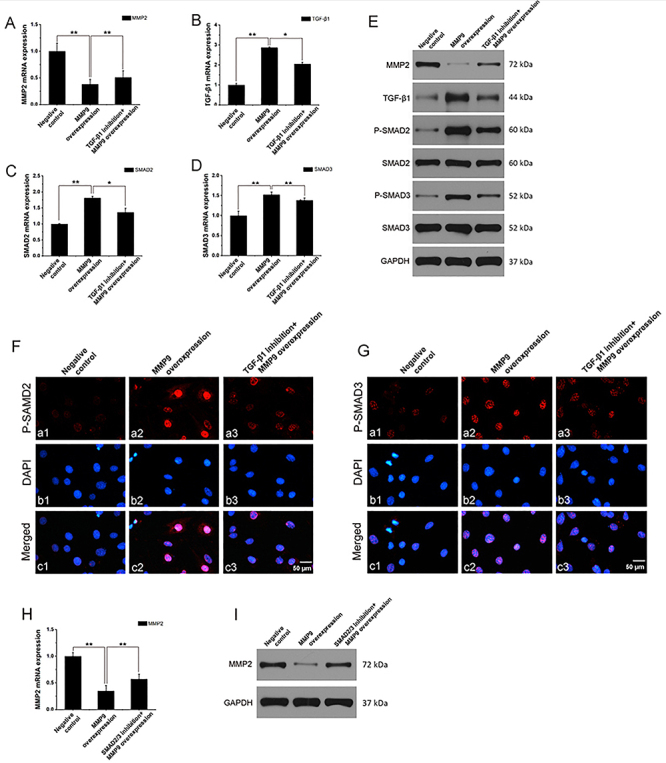
MMP9-regulated MMP2 expression was inhibited by TGF-β1 or SMAD2/3 in MC3T3-E1 cells. MC3T3-E1 cells were pretreated in the presence or absence of TGF-β1 or SMAD2/3 inhibitor for 1 h. After that, cells were transfected with pcDNA3.1 or pcDNA3.1-mMMP9 and cultured for 48 h. mRNA expression of (**A**) MMP2, (**B**) TGF-β1, (**C**) SMAD2, and (**D**) SMAD3 was measured by qRT-PCR. **E**, Protein expression of MMP2, TGF-β1, SMAD2, SMAD3, phospho-SMAD2, and phospho-SMAD3 was measured by western blot. Immunofluorescence analysis of (**F**) phospho-SMAD2 and (**G**) phospho-SMAD3 in MC3T3-E1 cells; scale bar 50 μm. **H**, mRNA and (**I**) protein expression of MMP2 was measured by qRT-PCR and western blot. The data are representative of three independent experiments and are reported as means±SD. *P<0.05, **P<0.01; ANOVA.

**Figure 3:**
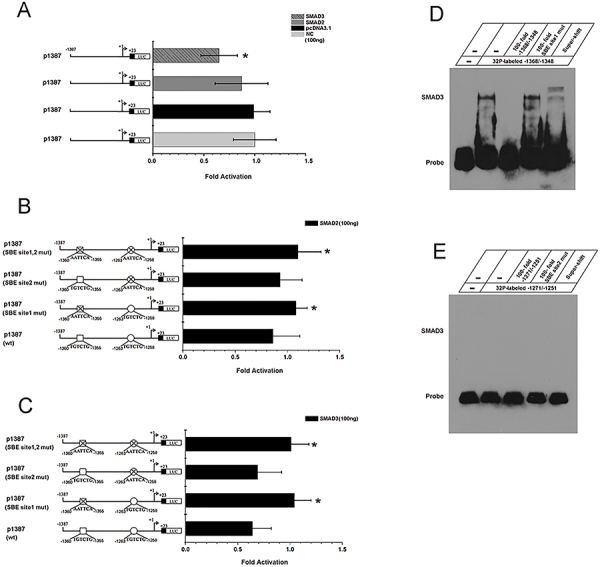
MMP9 downregulated *MMP2* gene transcription through SMAD2/3. **A**, pGL-Luc-mMMP2-1387/+23 (p1387) was co-transfected with 100 ng pcDNA3.1-SMAD3 or pcDNA3.1-SMAD3 or pCDNA3.1. Transcription results were computed as luciferase activities per mg of total protein. The value obtained from the control group was considered as 1-fold. Fold increases were calculated by dividing the individual value by the control group. **B**, p1387 Wt, p1387 site1 mut, p1387 site2 mut, or p1387 site1,2 mut were co-transfected with 100 ng pcDNA-Smad2. **C**, p1387 Wt, p1387 site1 mut, p1387 site2 mut, or p1387 site1,2 mut were co-transfected with 100 ng pcDNA-Smad3. The data are reported as means±SD from independent experiments in triplicate. **D**, SMAD3 bound to -1368/-1348 in *MMP2* promoter region. **E**, SMAD3 did not bind to -1271/-1251 in *MMP2* promoter region. ^32^P-labeled double stranded *MMP2* probes were incubated with 5 μg of SMAD3 overexpressed cell nuclear extracts. The sequences selected for *MMP2* probes are as follows: -1368 to -1348: (5'-CAAAGTCTTGTCTGAAGAGGA-3'), -1271 to -1251: (5'-AACCAGAATGTCTGATTTTTA-3'). Competition analysis of the SMAD3-MMP2 complex formation with 100-fold excess of unlabeled competitors or SBE site 1 mut (5'-CAAAGTCTAATTCAAAGAGGA-3') or SBE site 2 mut (5'- AACCAGAAAATTCAATTTTTA -3'). Data are reported as means±SD. *P<0.05; ANOVA.

**Figure 4:**
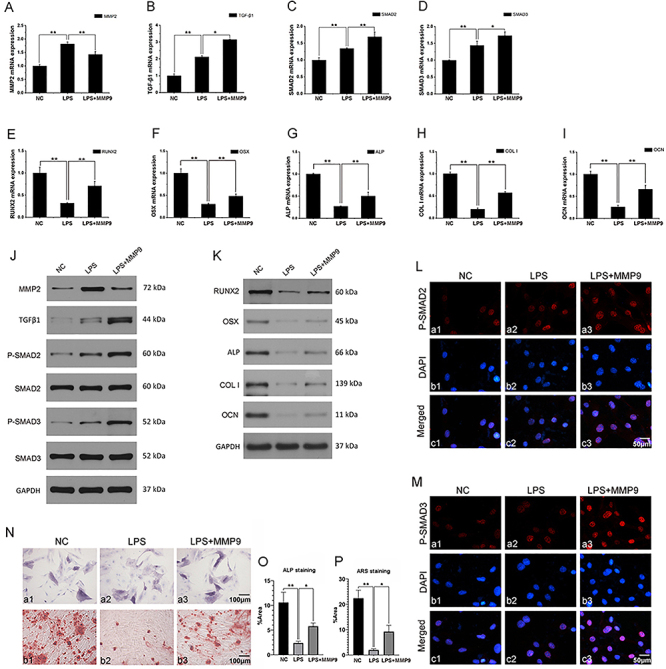
MMP9 regulated RUNX2, OSX, ALP, COL I, and OCN expression in lipopolysaccharide (LPS)-induced MC3T3-E1 cells. After pretreatment with pcDNA3.1 or pcDNA3.1-mMMP9 for 48 h, MC3T3-E1 cells were stimulated or not with 20 μg/mL LPS and cultured for another 12 h. mRNA expression of (**A**) MMP2, (**B**) TGF-β1, (**C**) SMAD2, (**D**) SMAD3, (**E**) RUNX2, (**F**) OSX, (**G**) ALP, (**H**) COL I, and (**I**) OCN was measured by qRT-PCR. **J**, Protein expression of MMP2, TGF-β1, P-SMAD2, SMAD2, P-SMAD3, and SMAD3 was measured by western blot. **K**, Protein expression of RUNX2, OSX, ALP, COL I, and OCN was measured by western blot. Immunofluorescence analysis of (**L**) phospho-SMAD2 and (**M**) phospho-SMAD3 in MC3T3-E1 cells; scale bar 50 μm. **N**, Alkaline phosphatase (ALP) staining (a1-a3) and alizarin red S (ARS) staining (b1-b3). **O**, Quantification of ALP staining. **P**, Quantification of ARS staining. Data are representative of three independent experiments and are reported as means±SD. *P<0.05, **P<0.01; ANOVA.

### MMP9 expression was increased by MMP9 DNA transfection and inhibited by MMP9 inhibitor

To assess the baseline expression of MMP9 during osteogenesis, MC3T3 cells were cultured under standard conditions in the absence of LPS. MC3T3-E1 cells were pretreated with or without MMP9 inhibitor for 1 h. After that, cells were transfected with pcDNA3.1 or pcDNA3.1-mMMP9 and cultured for 48 h. MMP9 production in the MMP9 overexpression group was significantly higher than in the control group at both mRNA and protein levels (P<0.01) (Figure 1A and G). MMP9 expression was inhibited by MMP9 inhibitor both at mRNA and protein levels (P<0.01) (Figure 1B and G).

### MMP9 inhibited MMP2 expression in MC3T3 cells

MC3T3-E1 cells were pretreated with or without MMP9 inhibitor for 1 h. After that, cells were transfected with pcDNA3.1 or pcDNA3.1-mMMP9 and cultured for another 48 h. MMP2 expression was measured by qRT-PCR and WB. At the mRNA level, MMP9 suppressed MMP2 expression (P<0.05) (Figure 1C). However, when the cells were pretreated with MMP9 inhibitor (20 μM) 1 h prior to MMP9 DNA transfection, MMP2 expression increased in the MMP9 inhibition group compared to the MMP9 DNA transfection group (P<0.05) (Figure 1C). At the protein level, MMP2 expression in MC3T3-E1 cells was much lower in the MMP9 overexpression group compared to the control group. There was a greater quantity of MMP2 expressed in the MMP9 inhibition group compared to the MMP9 overexpression group (Figure 1G).

### MMP9 promoted TGF-β1 expression as well as SMAD2/3 phosphorylation and nuclear translocation in MC3T3 cells

To determine whether MMP9 regulated TGF-β1 and SMAD2/3 expression, MC3T3-E1 cells were pretreated with or without MMP9 inhibitor for 1 h and for 48 h. After that, pcDNA3.1 or pcDNA3.1-mMMP9 was added to the medium, and the cells were cultured for another 48 h. qRT-PCR and WB were performed to measure TGF-β1 and SMAD2/3 expression. At the mRNA level, a significant increase in TGF-β1 expression was observed in the MMP9 overexpression group compared to the control group (P<0.01). However, when the cells were pretreated with MMP9 inhibitor (20 μM) for 1 h, TGF-β1 expression decreased compared to the MMP9 overexpression group (P<0.01) (Figure 1D). SMAD2 and SMAD3 expression in MC3T3 cells was higher in the MMP9 overexpression group compared to the control group (P<0.01), and SMAD2 and SMAD3 expression was slightly lower in the MMP9 inhibition group compared to the MMP9 overexpression group at the mRNA level (P<0.05) (Figure 1E and F).

At the protein level, TGF-β1 expression was much higher in the MMP9 overexpression group compared to the control group, and its expression decreased in the MMP9 inhibition group compared to the MMP9 overexpression group. No significant difference was observed in SMAD2 and SMAD3 expression among the negative control, MMP9 overexpression, and MMP9 inhibition groups. However, phospho-SMAD2 and phospho-SMAD3 expression in MC3T3 cells was higher in the MMP9 overexpression group compared to the control group. Phospho-SMAD2 and phospho-SMAD3 expression was lower in the MMP9 inhibition group compared to the MMP9 overexpression group (Figure 1G).

The immunofluorescence data indicated that phospho-SMAD2 and phospho-SMAD3 were significantly accumulated in the nuclei of MC3T3 cells after MMP9 treatment, whereas in the control group, phospho-SMAD2 and phospho-SMAD3 were distributed in the nucleus and cytoplasm. When the cells were pretreated with MMP9 inhibitor (20 μM) for 1 h, there was less phospho-SMAD2/3 accumulated in the nuclei (Figure 1H and I). These results demonstrated that the MMP9 inhibitor significantly inhibited the phosphorylation and nuclear translocation of SMAD2 and SMAD3.

### MMP9-regulated MMP2 expression and SMAD2/3 phosphorylation and nuclear translocation were inhibited by TGF-β1 inhibitor in MC3T3 cells

We further determined whether MMP9 regulated MMP2 and SMAD2/3 expression through TGF-β1. MC3T3 cells were pretreated with TGF-β1 inhibitor (20 μM) 1 h prior to MMP9 transfection for 48 h. As expected, at the mRNA level, MMP2 expression was higher in the TGF-β1 inhibition group compared to the MMP9 overexpression group (P<0.01) (Figure 2A). TGF-β1, SMAD2, and SMAD3 expression was lower in the TGF-β1 inhibition group compared to the MMP9 overexpression group (P<0.05) (Figure 2B-D). At the protein level, MMP2 expression was higher in the TGF-β1 inhibition group compared to the MMP9 overexpression group. TGF-β1 expression was lower in the TGF-β1 inhibition group compared to the MMP9 overexpression group. There was no significant difference in SMAD2 and SMAD3 expression among the negative, MMP9 overexpression, and TGF-β1 inhibition groups. However, expression of phospho-SMAD2 and phospho-SMAD3 was lower in the TGF-β1 inhibition group compared to the MMP9 overexpression group (Figure 2E). Immunofluorescence data demonstrated that the TGF-β1 inhibitor markedly reduced the phosphorylation and nuclear translocation of SMAD2 and SMAD3 (Figure 2F and G). These data demonstrated that the MMP9-mediated decreases in MMP2 expression and increases in SMAD2/3 expression were associated with TGF-β1 levels.

### MMP2 expression was regulated by SMAD2/3

We further determined whether MMP9 regulated MMP2 expression through SMAD2/3 by pretreating MC3T3 cells with SMAD2/3 inhibitor (20 μM) 1 h prior to MMP9 transfection for 48 h. As expected, at both the mRNA and protein levels, MMP2 expression was higher in the SMAD2/3 inhibition group compared to the MMP9 overexpression group (P<0.01) (Figure 2H and I).

The mouse *MMP2* gene regulatory region was analyzed by the Transcription Element Search System (TESS) (http://www.cbil.upenn.edu/cgi-bin/tess/tess) to search for potential SMAD binding elements (SBEs). In the proximal promoter of the *MMP2* gene, two SBEs were found (SBE site1: -1360 to -1355, SBE site2: -1263 to -1258). To clarify the biological functions of the two SBEs in the mouse *MMP2* promoter, the *MMP2* promoter -1387/+23 (p1387) was subcloned into the Luc-report vector (pGL-Luc-mMMP2-1387/+23). To determine of transcriptional activity, pGL-Luc-mMMP2-1387/+23 was transfected into 293T cells in the presence of pcDNA3.1-SMAD3 or pcDNA3.1-SMAD3 or pCDNA3.1 for 48 h. The results showed that SMAD2 and SMAD3 inhibited mMMP2-1387/+23 promoter activity (Figure 3A). To determine the biological functions of these two SBE sites, we generated three mutant DNA constructs: p1387 SBE site1-mut, p1387 SBE site2-mut, and p1387 SBE site1,2-mut. We found that the promoter activities of p1387 mutant constructs were higher than that of the p1387 Wt construct when 100 ng of either SMAD2 or SMAD3 existed (Figure 3B and C).

EMSA showed that the labeled double-stranded DNA sequence containing SBE site1 (-1368 to -1348: 5′-CAAAGTCTTGTCTGAAGAGGA-3′) was bound by SMAD3 overexpression cell nuclear extract *in vitro* (Figure 3D). However, the labeled double-stranded DNA sequence containing SBE site2 (-1271 to -1251: 5′-AACCAGAATGTCTGATTTTTA-3′) did not bind to SMAD3 in an overexpressed cell nuclear extract *in vitro* (Figure 3E). Competition assay showed the SMAD3 protein-DNA complex from *MMP2* promoter was competed away by 100-fold molar excess of the unlabeled homologous element. However, the affinity of the SMAD3 protein-DNA complex to bind to the *MMP2* promoter was much greater when there was a 100-fold molar excess of the unlabeled mutant element. We performed a super-shift assay using an anti-SMAD3 antibody. Incubation of the nuclear extracts with anti-SMAD3 antibody and the labeled double-stranded DNA containing SBE site1 led to the formation of slower migrating protein-DNA complexes (Figure 3D). These results verified that SMAD3 was able to bind to -1360 to -1355 in the *MMP2* promoter *in vitro*. However, it did not bind to -1263 to -1258 in the *MMP2* promoter *in vitro*.

### MMP9 regulated osteogenesis as well as MMP2 in LPS-induced MC3T3 cells

We next questioned whether this pro-osteogenic function is maintained in a pro-inflammatory microenvironment. After pretreatment with pcDNA3.1 or pcDNA3.1-mMMP9 for 48 h, MC3T3-E1 cells were stimulated or not with 20 μg/mL LPS for another 12 h. qRT-PCR and WB results showed that MMP2 decreased in the LPS+MMP9 group compared to that in the LPS group (P<0.01) (Figure 4A and J). However, TGF-β1 (P<0.05), phospho-SMAD2 (P<0.01), and phospho-SMAD3 (P<0.05) expression increased in the LPS+MMP9 group compared to that in the LPS group (Figure 4B-D and J). The immunofluorescence data demonstrated that MMP9 increased the phosphorylation of SMAD2 and SMAD3 (Figure 4L and M). These data demonstrated that MMP9 decreased MMP2 expression and increased TGF-β1 and SMAD2/3 expression in LPS-induced MC3T3-E1 cells.

qRT-PCR and WB were performed to measure RUNX2, OSX, ALP, COL I, and OCN expression. Treatment with LPS (20 μg/mL) decreased RUNX2, OSX, ALP, COL I, and OCN expression, and increased RUNX2, OSX, ALP, COL I, and OCN expression in the LPS+MMP9 group compared to that in the LPS group (P<0.01). The same results were obtained for qRT-PCR and WB analysis (Figure 4E-I and K).

The staining results showed that ALP expression decreased with LPS (20 μg/mL) stimulation. However, there was increased ALP expression in the LPS+MMP9 group compared to that in the LPS group (Figure 4N a1-a3 and O) (P<0.01). We also detected mineralization deposit MC3T3-E1 cells. The result showed that 20 μg/mL LPS reduced mineralization deposit. However, there was increased mineralization deposit in the LPS+MMP9 group compared to that in the LPS group (Figure 4N b1-b3 and P) (P<0.01).

## Discussion

MMP2 and MMP9 possess similar biological characteristics (13). It has been reported that they are important regulators for osteogenesis and osteolysis (19,20). In the present research, we found that MMP9 inhibited MMP2 expression in MC3T3-E1 cells. To clarify the signaling pathways in this process, we examined TGF-β1 and SMAD2/3 expression. The TGF-β family triggers signaling in cells via binding to the TGF-β receptor complex and activating or repressing gene expression via Smad-dependent or Smad-independent signaling pathways (21). Studies have demonstrated that TGF-β activates MMPs via different signaling pathways. Lamar et al. (22) reported that TGF-β activated the expression of MMP9 binding with α3β1. Sinpitaksakul et al. (23) found that TGF-β1 signaling induced MMP9 expression via Smad and mLCK. Gweon and Kim (24) and Takahashi et al. (25) reported that TGF-β1 induced MMP9 upregulation in pericytes via p38 mitogen-activated protein (MAP) kinase signaling. Kim et al. (26) found that TGF-β activated the expression of MMP-2 via ATF-2. Additionally, Kobayashi et al. (27) reported that MMP9 activated TGF-β in fibroblasts. In the present study, we clarified that MMP9 inhibited MMP2 expression through TGF-β1-SMAD2/3 signaling in MC3T3-E1 cells *in vitro*.

We identified two potential SMAD2/3 binding sites in the *MMP2* gene promoter region using TESS (University of Pennsylvania, USA) and DNA-protein binding assays. Dual luciferase assay demonstrated that SMAD2/3 decreased *MMP2* gene promoter activity via the two SBEs. Mutations of SBEs in the *MMP2* gene promoter increased *MMP2* gene transcription. We next performed EMSA and found that SMAD3 was able to bind to the promoter region of *MMP2 in vitro*. We further confirmed these results by Supershift and Competition assays.

The function of MMPs is much more complex and subtle than straightforward demolition. MMPs are effectors and regulators in normal growth and development as well as in pathological processes. In addition to the ability to degrade the ECM, MMPs also regulate the extracellular environment by affecting cellular behavior. In the present research, we analyzed the expression of osteogenic markers RUNX2, OSX, ALP, COL I, and OCN. ALP is an enzyme secreted by osteoblasts. It is usually used as one of the indicators to evaluate the mineralization ability of osteoblasts (28,29). ALP expression was found increased by MMP9 in 20 μg/mL LPS-induced osteoblasts (7). RUNX2, also known as core-binding factor α1, is an important member of the Runt family. It plays a critical role not only in osteoblast differentiation but also in osteoblast function, by acting as a positive regulator that upregulates the expression of bone matrix proteins, including OCN and type I collagen ([Bibr B30] [Bibr B31] [Bibr B32] 3033). OSX is an osteoblast-specific transcription factor that is critical for bone formation (34,35). OCN is a non-collagenous protein secreted by osteoblasts in the late stage of differentiation (36). RUNX2, OSX, ALP, COL I, and OCN expressions were found increased by MMP9 in LPS-induced osteoblasts. These results suggest that MMP9 regulates osteogenesis by promoting bone formation, consistent with the finding that MMP9 increased OPG and OCN expression in LPS-stimulated cells (7).

## Conclusion

We found that MMP9 activated the expression of TGF-β1 and phosphorylation of SMAD2/3. Phosphorylated SMAD2/3 translocalized into nuclei and was able to bind to SBEs in the *MMP2* gene promoter, inhibiting *MMP2* gene transcription. Additionally, we confirmed that MMP9 increased RUNX2, OSX, ALP, COL I, and OCN expression in LPS-induced MC3T3 cells. Therefore, MMP9 may regulate osteogenesis through the TGF-β1-SMAD2/3/-MMP2 signaling pathway during the inflammation process. This study helps reveal the complex molecular mechanisms among MMPs in the process of inflammation and provides an experimental and theoretical basis for the prevention and treatment of inflammation.

## Data Availability

All data generated or analyzed during this study are included in this published article.

## References

[1] Somerville RPT Oblander SA, Apte SS (2003). Matrix metalloproteinases: old dogs with new tricks. Genome Biol.

[2] Holliday LS, Welgus HG, Fliszar CJ, Veith GM, Jeffrey JJ, Gluck SL (1997). Initiation of osteoclast bone resorption by interstitial collagenase. J Biol Chem.

[3] Delaissé JM, Engsig MT, Everts V, del Carmen Ovejero M, Ferreras M, Lund L (2000). Proteinases in bone resorption: obvious and less obvious roles. Clin Chim Acta.

[4] Vu TH, Shipley JM, Bergers G, Berger JE, Helms JA, Hanahan D (1998). MMP-9/gelatinase B is a key regulator of growth plate angiogenesis and apoptosis of hypertrophic chondrocytes. Cell.

[5] Mosig RA, Dowling O, DiFeo A, Ramirez MC, Parker IC, Abe E (2007). Loss of MMP-2 disrupts skeletal and craniofacial development and results in decreased bone mineralization, joint erosion and defects in osteoblast and osteoclast growth. Hum Mol Genet.

[6] McQuibban GA, Gong JH, Tam EM, McCulloch CA, Clark-Lewis I, Overall CM (2000). Inflammation dampened by gelatinase A cleavage of monocyte chemoattractant protein-3. Science.

[7] Zhang H, Liu L, Jiang C, Pan K, Deng J, Wan C (2020). MMP9 protects against LPS-induced inflammation in osteoblasts. Innate Immun.

[8] Nagase H (1997). Activation mechanisms of matrix metalloproteinases. Biol Chem.

[9] Sato H, Takino T, Okada Y, Cao J, Shinagawa A, Yamamoto E (1994). A matrix metalloproteinase expressed on the surface of invasive tumour cells. Nature.

[10] Knäuper V, Will H, López-Otin C, Smith B, Atkinson SJ, Stanton H (1996). Cellular mechanisms for human procollagenase-3 (MMP-13) activation. Evidence that MT1-MMP (MMP-14) and gelatinase a (MMP-2) are able to generate active enzyme. J Biol Chem.

[11] Dreier R, Grässel S, Fuchs S, Schaumburger J, Bruckner P (2004). Pro-MMP-9 is a specific macrophage product and is activated by osteoarthritic chondrocytes via MMP-3 or a MT1-MMP/MMP-13 cascade. Exp Cell Res.

[12] Steenport M, Khan KM, Du B, Barnhard SE, Dannenberg AJ, Falcone DJ (2009). Matrix metalloproteinase (MMP)-1 and MMP-3 induce macrophage MMP-9: evidence for the role of TNF-alpha and cyclooxygenase-2. J Immunol.

[13] Visse R, Nagase H (2003). Matrix metalloproteinases and tissue inhibitors of metalloproteinases: structure, function, and biochemistry. Circ Res.

[14] Wan CY, Yuan GH, Yang JW, Sun Q, Zhang L, Zhang J (2014 May). MMP9 deficiency increased the size of experimentally induced apical periodontitis. J Endod.

[15] Zou ML, Chen ZH, Teng YY, Liu SY, Jia Y, Zhang KW (2021). The smad dependent TGF-beta and BMP Signaling pathway in bone remodeling and therapies. Front Mol Biosci.

[16] Chen G, Deng C, Li YP (2012). TGF-beta and BMP signaling in osteoblast differentiation and bone formation. Int J Biol Sci.

[17] Schmittgen TD, Livak KJ (2008). Analyzing real-time PCR data by the comparative C(T) method. Nat Protoc.

[18] Chen S, Unterbrink A, Kadapakkam S, Dong J, Gu TT, Dickson J (2004). Regulation of the Cell Type-specific dentin sialophosphoprotein gene expression in mouse odontoblasts by a novel transcription repressor and an activator CCAAT-binding factor. J Biol Chem.

[19] Martignetti JA, Aqeel AA, Sewairi WA, Boumah CE, Kambouris M, Mayouf SA (2001). Mutation of the matrix metalloproteinase 2 gene (MMP2) causes a multicentric osteolysis and arthritis syndrome. Nat Genet.

[20] Wan CY, Yin Y, Li X, Liu MM, Goldman G, Wu LA (2024). MMP-9 deficiency accelerates the progress of periodontitis. Genes Dis.

[21] Thielen NGM, van der Kraan PM, van Caam APM (2019). TGFbeta/BMP signaling pathway in cartilage homeostasis. Cells.

[22] Lamar JM, Iyer V, DiPersio CM (2008). Integrin alpha3beta1 potentiates TGFbeta-mediated induction of MMP-9 in immortalized keratinocytes. J Invest Dermatol.

[23] Sinpitaksakul SN, Pimkhaokham A, Sanchavanakit N, Pavasant P (2008). TGF-beta1 induced MMP-9 expression in HNSCC cell lines via Smad/MLCK pathway. Biochem Biophys Res Commun.

[24] Gweon EJ, Kim SJ (2014). Resveratrol attenuates matrix metalloproteinase-9 and -2-regulated differentiation of HTB94 chondrosarcoma cells through the p38 kinase and JNK pathways. Oncol Rep.

[25] Takahashi Y, Maki T, Liang AC, Itoh K, Lok J, Osumi N (2014). p38 MAP kinase mediates transforming-growth factor-beta1-induced upregulation of matrix metalloproteinase-9 but not -2 in human brain pericytes. Brain Res.

[26] Kim ES, Sohn YW, Moon A (2007). TGF-beta-induced transcriptional activation of MMP-2 is mediated by activating transcription factor (ATF)2 in human breast epithelial cells. Cancer Lett.

[27] Kobayashi T, Kim H, Liu X, Sugiura H, Kohyama T, Fang Q (2014). Matrix metalloproteinase-9 activates TGF-beta and stimulates fibroblast contraction of collagen gels. Am J Physiol Lung Cell Mol Physiol.

[28] Kim HK, Cho SG, Kim JH, Doan TK, Hu QS, Ulhaq R (2009). Mevinolin enhances osteogenic genes (ALP, type I collagen and osteocalcin), CD44, CD47 and CD51 expression during osteogenic differentiation. Life Sci.

[29] Liu XW, Ma B, Zi Y, Xiang LB, Han TY (2021). Effects of rutin on osteoblast MC3T3-E1 differentiation, ALP activity and Runx2 protein expression. Eur J Histochem.

[B30] Komori T (2017). Roles of runx2 in skeletal development. Adv Exp Med Biol.

[B31] Ducy P, Zhang R, Geoffroy V, Ridall AL, Karsenty G (1997). Osf2/Cbfa1: a transcriptional activator of osteoblast differentiation. Cell.

[B32] Prince M, Banerjee C, Javed A, Green J, Lian JB, Stein GS (2001). Expression and regulation of Runx2/Cbfa1 and osteoblast phenotypic markers during the growth and differentiation of human osteoblasts. J Cell Biochem.

[33] Ducy P, Starbuck M, Priemel M, Shen J, Pinero G, Geoffroy V (1999). A Cbfa1-dependent genetic pathway controls bone formation beyond embryonic development. Genes Dev.

[34] Wang C, Liao H, Cao Z (2016). Role of osterix and microRNAs in bone formation and tooth development. Med Sci Monit.

[35] Zhang C (2012). Molecular mechanisms of osteoblast-specific transcription factor Osterix effect on bone formation. Beijing Da Xue Xue Bao Yi Xue Ban.

[36] Neve A, Corrado A, Cantatore FP (2013). Osteocalcin: skeletal and extra-skeletal effects. J Cell Physiol.

